# Combined Preventive and Preconditioning Treatments for the Comorbidity of Alzheimer’s Disease and Ischemic Stroke in a GluN3A Knockout Mouse and a 5xFAD Mouse

**DOI:** 10.3390/cells14231871

**Published:** 2025-11-26

**Authors:** Shan Ping Yu, Xiaohuan Gu, Michael Q. Jiang, Ananth Sastry, Lingyue Wu, Yiying Li, Ling Wei

**Affiliations:** 1 Department of Anesthesiology, Emory University School of Medicine, 101 Woodruff Circle, Suite 617, Atlanta, GA 30322, USA; xiaohuan.gu@emory.edu (X.G.); michaeljiang@emory.edu (M.Q.J.); ananth.sastry@emory.edu (A.S.); lingyue.wu@emory.edu (L.W.); lyy050722@gmail.com (Y.L.); lwei7@emory.edu (L.W.); 2 Center for Visual & Neurocognitive Rehabilitation, Atlanta VA Medical Center, Decatur, GA 30033, USA

**Keywords:** Alzheimer’s disease, NMDA receptors, GluN3A subunit, preclinical stage, ischemic stroke, preemptive treatment, preconditioning effect, memantine, neuroprotection, amyloid-independent, functional protection

## Abstract

Alzheimer’s disease (AD) and stroke have been identified as risk factors for each other. More than half of AD patients suffer stroke attacks and worse ischemic injuries. There has been a lack of research focus and clinical treatment for the comorbidity of these neurological disorders. AD and ischemic stroke share characteristic pathophysiology, including hyperactivities of excitatory neurons and NMDA receptors (NMDARs), excitotoxicity, and synapse/neurovascular destruction. Our recent investigations identified the deficiency of the NMDAR regulatory GluN3A (NR3A) subunit as a novel pathogenesis of sporadic AD. The present investigation tested a preemptive treatment to prevent AD development in two AD models and, in the meantime, to prime the susceptible brain against upcoming ischemic attacks. In the preclinical stage of 3-month-old GluN3A KO mice, an NMDAR-mediated sporadic AD model, and 5xFAD mice, an amyloid-based familial AD model, treatments with memantine (MEM), an NMDAR antagonist (10 mg/kg/day in drinking water) and a drug-free control were started when cognition of these mice was generally normal. Three months later, the mice were subjected to focal cerebral ischemic surgery, followed by continued 1.5–2.0 months of MEM or vehicle control. Morphological, pathological, and functional assessments were performed and compared at different time points. In both AD models, the early MEM treatment confined AD progression before and after stroke, reduced ischemia-induced brain injury, suppressed neuroinflammation, and improved locomotion, sensorimotor, psychological, and cognitive functions. This is the first report endorsing a shared mechanism of NMDAR hyperactivity in AD and stroke in AD models with distinctive risk factors. The dual therapeutic effects of the preemptive MEM treatment provide a disease-modifying possibility for individuals who are susceptible to sporadic or familial AD as well as ischemic stroke.

## 1. Introduction

Alzheimer’s disease (AD) and ischemic stroke are common neurological disorders; both are leading causes of disability and death in aging populations [1]. Over half of AD patients encounter one or more stroke attacks and they usually have more severe outcomes, such as higher mortality rates than other patients [2,3]. Within three years after an ischemic attack, a third of stroke patients develop post-stroke dementia (PSD). In chronic phases, both AD and stroke involve programmed cell death, sensorimotor/olfactory deficits, psychological alterations, cognitive decline, and β-amyloid (Aβ) deposition [1,3,4]. A longitudinal cohort study compared 12,629 ischemic stroke cases from 2007–2017, identifying PSD as an independent predictor of patient mortality [5]. Thus, AD/AD-related dementia (ADRD) and stroke are risk factors for each other and often coexist [2,5]. However, the mechanism behind their relationship and their interactions are unclear. Up to now, stroke and AD have usually been studied in unconnected research fields and viewed as distinctive acute and chronic neurological disorders. More recent evidence, however, revealed that AD and stroke share a number of hallmark pathophysiological features [4,6]. These may include, but not limited to, hyperactivities of excitatory neurons underlined by overactivation of glutamatergic N-methyl-D-aspartate (NMDA) receptors (NMDARs), increases in intracellular free Ca^2+^ ([Ca^2+^]_i_), energy metabolism disruptions, excitotoxic and programmed neuronal loss, neuroinflammation, synaptic and neural network impairments, neurovascular breakdown, Aβ/tau pathology, and progression of psychological/cognitive symptoms [1,4,7]. After several decades of investigations in each research field, numerous neuroprotective and anti-amyloid drug/antibody therapies have been developed. In clinical trials, however, the inconsistency or lack of functional benefits have hampered their clinical translation, which may be attributed to several dilemmas and obstacles [8,9]. The failure in therapy development for common clinical cases, such as sporadic AD and ischemic stroke, could be affected by, at least partly, the knowledge gap in basic research and clinical studies on fundamental and shared pathological mechanisms and the comorbidity of these two neurological disorders.

A major early advance in neuroscience research was the identification of glutamate-mediated excitotoxicity via NMDAR overactivation [10]. NMDAR-mediated and Ca^2+^-induced excitotoxicity was originally revealed in neuronal cell cultures of excitotary death and animal models of ischemic stroke [11,12,13,14]. Later on, neuronal and NMDAR hyperactivities were observed in the slow progression of AD and ADRD in animal models and human patients [15,16]. Although the alteration seems trivial and does not cause immediate cell death and acute tissue injury as in ischemic stroke, the Ca^2+^ hypothesis of AD suggests that small but sustained increases of [Ca^2+^]_i_ can be responsible for chronic neurodegeneration. The enduring stress cascade ultimately leads to neuronal damage mediated by Ca^2+^-activated, deleterious signals [15,16,17].

Currently, there are a few clinical treatments for ischemic stroke, such as recombinant tissue plasminogen activator (rTPA) and endovascular thrombectomy, to restore local cerebral blood flow (LCBF) [18]. Both treatments must be administered within a limited time scale (hours) after the stroke. While a powful preconditioning protection can be achieved by sublethal hypoxia, ischemia and many chemicals/reagents, the required pretreatment makes it impractical in clinical settings [19]. In the field of AD research, cholinesterase inhibitors and the NMDAR antagonist memantine (MEM) are FDA-approved symptomatic treatments for moderate-to-severe AD patients. However, their clinical efficacy varies and remains to be improved with a better understanding of AD pathogenesis [20].

The prolonged progression of AD involves three clinical stages, based on the occurrence and severity of cognitive deficits [21]. The preclinical stage shows early neurodegenerative symptoms such as psychological and sensory/olfactory deficits while cognition is basically still normal. The preclinical stage of initial AD pathophysiology in humans evolves for 10–25 years in susceptible individuals before clinical diagnosis of mild cognitive impairment (MCI)/AD [22]. This “silent” stage, preceding cognitive symptoms in adulthood, represents an extended period of original neuropathogenesis and a wide time window of opportunity for disease-modifying interventions. Currently, the mechanisms by which NMDAR activity is persistently upregulated during the preclinical stage and its link to AD progression remain obscure. A few investigations in this research area have focused only on the NMDAR GluN1 and GluN2 subunits. For instance, increased GluN2B expression was suggested to mediate Aβ-induced NMDA hyperactivity [23]. AD research extensively studies the Aβ hypothesis in rodents. It is proposed that neuronal and NMDAR overactivation are consequences of amyloid toxicity, both in vitro systems and in transgenic AD models [24]. This popular concept has been disputed by preclinical and clinical observations that neuronal and NMDAR hyperactivities often exist in the absence of amyloid deposition [17,25,26,27,28,29].

In the human brain, the onset age of Aβ deposition is approximately 50 years old [30]. Meanwhile, noticeable sensorimotor and psychological abnormalities, such as olfactory deficits, usually emerge in the preclinical stage at younger ages [31,32]. The main cause for the abnormal Aβ production, neurodegeneration, and functional changes in neurological disorders such as late-onset sporadic AD is likely complicated [33]. Genetic influences [30] and ideas like “cognitive reserve” [34] have been suggested. Aside from exceedingly delineated molecular mechanisms of amyloid peptide metabolism, there has been little information on the foremost trigger during the year/decade-long process before endogenous amyloid pathology. Alternative or additional mechanisms could contribute to the initial pathogenesis, pathophysiology, and pathology during the preclinical stage of sporadic AD/ADRD.

Our investigation discovered that the deficiency of GluN3A (NR3A), an NMDAR regulatory subunit, has a chronic pathogenic effect [17,35,36]. It causes mild hyperactivity of NMDARs, leading to dysregulation of Ca^2+^ homeostasis, neuronal inflammation, synaptic disruption, and degenerative excitotoxicity. GluN3A knockout (KO) mice display multiple early and late syndromes, including age-dependent sensory deficits, psychological/psychiatric disorders, and cognitive declines [17], the syndrome and progression closely mirror those found in AD patients [37,38,39]. Without transgenic expression of exogenous FAD genes or manipulation of amyloid metabolism, dementia spontaneously developed in the GluN3A KO mouse around 5–6 months of age. Endogenous Aβ deposition and tau hyperphosphorylation spontaneously emerge weeks to months after cognitive decline in these mice. These features point to an amyloid-independent AD mechanism and provide a unique sporadic AD model. The GluN3A KO mouse is especially suitable for studying the early, primary pathogenic events upstream of endogenous Aβ/tau pathology.

AD and ischemic stroke may have similar excitotoxicity through NMDAR/Ca^2+^ pathways, although the time courses are different. We suggest that a preventive therapy targeting the chronic pathogenic mechanism of AD simultaneously prepares the brain against acute excitotoxicity caused by unpredictable ischemic attacks. Recent progress has elucidated the distinct roles of synaptic NMDARs (sNMDARs) and extrasynaptic eNMDARs in plasticity, neuroprotection and excitotoxicity [4,40,41]. MEM preferably acts on eNMDARs to block excitotoxicity and is a clinically suitable NMDAR antagonist for chronic applications [4]. Notably, the GluN3A subunit is primarily located at extrasynaptic sites [4]. We recently provided evidence of the anti-AD efficacy of MEM in the preclinical stage of GluN3A KO and 5xFAD mice [17,36,42]. The current investigation examined the early MEM treatment for its anti-AD effect, as well as its preconditioning anti-stroke effect, in the GluN3A KO mouse as a sporadic AD model, and the 5xFAD mouse as a human amyloid pathology model. The results from these two AD models of diverse risk factors support that the preemptive approach is an effective preventive and preconditioning strategy for AD patients who are more vulnerable to stroke attacks during aging.

## 2. Materials and Methods

### 2.1. Animals, Animal Care, and Euthanasia Methods

GluN3A KO mice, 5xFAD mice, and their corresponding controls of WT adult mice (3–8 months old, male) were examined. A total of 151 mice were tested and analyzed in this investigation, including a 3% mortality rate due to disease progression and surgery procedures. Dead animals were excluded from data analysis, which is specified in the figure legend. There is no other exclusion in data analysis.

The GluN3A KO mouse was obtained from Drs. Nobuki Nakanishi and Stuart A. Lipton at Sanford-Burnham Medical Research Institute (La Jolla, CA, USA). Information of the background of GluN3A modified mice is available in published papers [43]. 5xFAD mice and breeding pairs were purchased from the Jackson Laboratory (Bar Harbor, ME, USA). These transgenic mice overexpress several mutant human proteins, including amyloid β (A4) precursor protein 695 (APP) with the Swedish, Florida, and London FAD mutations, and presenilin 1 (PS1) harboring two FAD mutations, M146L and L286V [44]. Mice produced from the breeding procedure resulted in heterozygous, homozygous or WT phenotypes, which we identified and verified using the genome typing method [45,46]. Adult male homozygous and WT mice were selected as AD and control mice. All mice were kept in standard cages placed in a dedicated room with a 12 h light and 12 h dark cycle at the Emory University animal facility. The room temperature was maintained at 22 ± 1 °C. Littermate WT and KO animals were used in control and experimental groups, respectively, following the randomization principles.

### 2.2. Memantine Treatment

GluN3A KO and 5xFAD mice received MEM (Sigma-Aldrich, St Louis, MO, USA); the company catalog number: PHR1886-1G; 10 mg/kg per day added to drinking water) or vehicle control at 3 months old, and the treatment lasted for the next 3 months before undergoing focal cerebral ischemia surgery. The MEM dosage was selected based on its efficacy and few side effects, consistent with clinical use [42,47]. The amount of water taken by animals was monitored to ensure the average daily dose by adjusting the drug concentration. To achieve the final dosage of 10 mg/kg/day, 0.25 mg/day is needed for a 25 g mouse (MEM solubility in water is 1 mg/mL). The equivalent drug dosage per day was calculated and the average drug concentration in drinking water was adjusted to ensure accurate daily intake. No maneuver was taken to influence the drinking behavior of mice. After the ischemic surgery, the animals received continuous MEM or vehicle treatments for an additional 1.5–2.0 months. Morphological, pathological, and functional inspections were carried out before and after stroke in the same or different study groups (Supplemental Scheme S1).

### 2.3. Cerebral Focal Ischemic Stroke Model of the Mouse

The cerebral focal ischemic insult specifically ligates the distal branches of the middle cerebral artery (MCA) supplying the right sensorimotor cortex [48]. Animals were placed on a temperature-controlled heating pad; the animal’s core temperature was monitored and maintained at 37.0  ±  0.5 °C. They received anesthesia with ketamine (80–100 mg/kg)/xylazine (5–10 mg/kg). Craniotomy was created via the right parietal skull and the transparent dura mater above the sensorimotor/barrel cortex was kept intact. Performed under a dissecting microscope, 10-0 sterile silk sutures were placed through the dura to occlude right side MCA branches. To significantly reduce the blood supply to the sensorimotor cortex, the common carotid artery (CCA) was additionally ligated for 7 min. Next, the CCA was exposed via a separate incision made in the neck, and it was transiently ligated by sterile 5-0 silk sutures. The suture of CCA was removed after the transient ligation. This unique focal ischemia model mimics many stroke cases of permanent vessel occlusion with spontaneous or drug-induced, e.g., tPA, partial reperfusion [49].

### 2.4. Local Cerebral Blood Flow Measurement (LCBF)

Under anesthesia, in vivo LCBF in the penumbra region of the cortex was surveyed using the Periscan system before and 3 days after the stroke surgery. Before the measurement, an incision was cut to expose the brain skull over the supplying territory of the right MCA. The laser probe was placed and centered over the right coronal suture. This scanner can measure LCBF in an area of 2.4  ×  2.4 mm^2^, which is an advantage over the conventional Laser Doppler method, which measures only a small point of blood flow. The value of LCBF was calculated from 6 repeated readings in each mouse. Data were collected and analyzed using the LDPI Win 2 software (Perimed AB, Stockholm, Sweden).

### 2.5. Animal Scarification Method

Animals were sacrificed under euthanasia at specified time points. For perfusion-fixed brain samples, the animal was positioned on the platform with its head down. Injection of Euthasol (pentobarbital sodium, 390 mg/mL) was done via IP into the lower right abdomen. The animal was observed for 30 s to ensure the disappearance of respiration and heartbeat. For experiments of Western blot analysis and immunostaining assessments, carbon dioxide (CO_2_) inhalation was employed for euthanasia, which has been widely used as a standard method for rodents. In this procedure, the mouse in a clear euthanasia chamber was visualized and the chamber was filled gradually with a gas flowmeter system following the Euthanasia Guidelines of the American Veterinary Medical Association. The final CO_2_ flow rate was kept for 1 min after the respiratory and heart rates of the animal stopped. The use of the automated machine and process were approved by the institutional IACUC (see above). After euthanasia, physical methods such as decapitation, thoracotomy, cervical dislocation, or terminal necropsy were carried out according to the approved protocol and experimental design.

### 2.6. Ischemia-Induced Infarct Volume Assessments

Three days after ischemic stroke, mouse brains were dissected according to the animal protocol. Brain samples were sliced into 1 mm-thick coronal sections using a mouse brain matrix (Harvard Bioscience, South Natick, MA, USA). The brain sections were placed in 2% 2,3-5-triphenyl-tetrazolium chloride solution (TTC) for 5 min. The brain section with TTC-positive and TTC-negative regions was scanned using a high-resolution HP scanner. The TTC-positive versus TTC-negative areas of six brain slides per animal, as well as the area of the ipsilateral and contralateral hemispheres, were measured using the NIH ImageJ software (version 1.54p; NIH, Bethesda, MD, USA). The infarct volume was calculated using the published indirect method [50].

### 2.7. Immunostaining and TUNEL Assays

Mice were sacrificed 3 days after ischemic stroke (~6 months of age) for morphological and pathological inspections. Brain samples were fixed in 10% formalin and NIHsliced into 10-μm-thick coronal sections using a cryostat vibratome (Leica CM 1950; Leica Microsystems, Buffalo Grove, IL, USA). A slide warmer was used for dehydration for 10 min, followed by a 10-mimute fixation in 10% buffered formalin phosphate solution. The brain sections was placed in an ethanol:acetic acid (2:1) solution for 10 min and washed out with PBS 3 times with 5 min incubation each. Triton-X 100 (0.2%, G-Biosciences, St. Louis, MO, USA; Cat#: 786-513) was applied for 10 min followed by 3 washes with PBS (5 min each). Fish gelatin (1%; Sigma-Aldrich, catalog# G7041) was used as the blocking solution for 1 h at room temperature. Brain sections were then incubated with primary antibodies diluted in PBS for 10 to12 h at 4 °C. The primary antibodies used in this investigation include: mouse anti-NeuN (1:2000, Abcam, Cambridge, UK; catalog# ab104224-1001), rabbit anti-amyloid β42 (1:400, Cell signaling, Danvers, MA, USA; catalog# 8243), chicken anti-GFAP (1:2000, Invitrogen, Carlsbad, CA, USA; catalog# PA1-10004), or rabbit anti-iba1 (1:2000, Abcam catalog# ab178847-1001). Sections were washed with PBS and incubated with corresponding secondary antibodies (Cy3, Alexa Fluor488, Cy5 goat/donkey anti-mouse, chicken, rabbit 1:500, Jackson ImmunoResearch Lab. West Grove, PA, USA) for 2 h. Sections were washed again 3 times with PBS then cover-slipped with the Vectashield mounting media with DAPI (Vector labs, Newark, CA, USA; catalog# H1200). Photographs were generated under a fluorescent microscope (BX51, Olympus, Tokyo, Japan) for next off-line image analysis. To detect cell death, the fluorometric TUNEL (terminal deoxynucleotidyl transferase biotin-dUPT nick-end labeling) kit was used, and the procedure followed the commercial guidelines provided by the company.

### 2.8. Western Blotting Analysis

Brain tissue samples were collected from the peri-infarct region of the ischemic cortex six weeks after stroke. Tissues were homogenized in a protein lysis buffer to extract proteins. The buffer solution contained: 20 mM Na_4_P_2_O_7_, 10 mM Tris-HCl (pH 7.4), 100 mM NaCl, 1 mM EDTA (pH 8.0), 1 mM EGTA, 2 mM Na_3_VO_4_, 1% Triton, and 1% protease inhibitor cocktail (Sigma-Aldrich, catalog# P8849). Thirty microgram protein from each tissue sample was loaded into a gradient gel (6%–20%) and run at a constant current until protein markers were adequately separated. Protein was then transferred onto polyvinyl difluoride membranes, which were next probed using a standard protocol. The membranes were then blocked with 5% bovine serum albumin in 1% TBST for 1 h and incubated overnight at 4 °C with primary antibodies, including: IL-6 (mouse 1:1000; Cell Signaling 12912S), IL-10 (rabbit 1:1000; Cell Signaling 12163S), IL1-beta (rabbit 1:1000; Cell Signaling 12703S), TNF-alpha (rabbit 1:1000; Cell Signaling 11948S), beta-Tubulin (rabbit 1:1000; Cell Signaling 2146S), BCL-2 (rabbit 1:1000; Cell Signaling 3498S), BDNF (rabbit 1:1000; Cell Signaling 47808S), GSK-3β (rabbit 1:1000; Cell Signaling 9315S), P-GSK-3β (rabbit 1:1000; Cell Signaling 9336S), P-CREB (rabbit 1:1000; Cell Signaling 9198S), Caspase-3 (rabbit 1:1000; Cell Signaling 9662S), and β-actin (mouse 1:2500; Sigma-Aldrich). After 3 washes with 0.1% TBST, the membranes were treated with ammonium persulfate (AP)-conjugated secondary antibodies (Promega, Madison, WI, USA) at room temperature for 2 h. The membranes were then washed in the 0.1% TBST solution for 2 h, and protein bands were developed using the nitro-blue tetrazolium and 5-bromo-4-chloro-3′indolyphosphate. The intensity of protein bands was quantified and subtracted from the background using the NIH ImageJ software (Bethesda, MD, USA). The ratio of the level of each target protein was normalized versus the loading control of the β-actin band.

### 2.9. Locomotion and Sensorimotor Functional Tests

Animals received different functional or behavioral tests at 3 months old (with a few days of variation) before ischemic stroke and 1.5–2.0 months after stroke. All animals were placed in the testing room and habituated for 30 min before testing.

The rotarod test assessed motor coordination and balance function on an accelerating rotarod (UGO Basile, Collegeville, PA, USA). Rotation of the rotarod began at a speed of 4 rotations per minute (rpm) and accelerated to 40 rpm over 5 min. Prior to ischemic stroke, mice were trained on the rotarod for 3 trials/day for 3 days; each trial lasted 5 min and was separated by 2 h intervals. Three days after the stroke, the time the animal remained on the beam was measured.

The adhesive dot removal test inspected the sensorimotor neuronal network and corresponding functional activities. A small adhesive dot was put on the testing mouse’s forepaws. The time that the mouse took to remove each sticker was recorded. All animals were pre-tested three times on the day before stroke to ensure they had normal sensorimotor responses. We paused recording when the animal failed to feel and contact the sticker within the first 2 minutes. The test was performed three times per mouse, and the average time was calculated.

### 2.10. Social Behavior and Psychological Tests

Three-chamber sociability and social novelty tests were employed to exam social affiliation and social memory. The testing mouse was initially allowed to freely explore the three-chamber box for 10 min. The social behavior test was performed by introducing a littermate mouse, covered by a wire cup, into one of the side chambers for 10 min. In the social novelty test, an unfamiliar mouse was introduced into the chamber. The familiar littermate mouse was then put into the different side of the box. The time the animal spent sniffing familiar and novel mice was recorded and analyzed.

The novel object test assessed social activity and recognition memory. During the training session, the mouse explored a box with two identical objects placed in adjacent corners for 10 min. After a retention session of one hour after training, the mice explored for another 10 min in the box contained a new object that replaced a previous identical one. The time of the mouse spent exploring the novel object and the familiar object was measured and the ratio was calculated.

The open field test evaluated anxiety-like behavior. The animal was placed into a box containing a 50 × 50 × 50 cm open field area, and allowed free movements for 10 min. The travel time and distance were monitored using the TopScan Clever System (Clever Sys, Inc., Reston, VA, USA), and the video recordings were examined and quantified by the TopScan Realtime Option Version 3.0 (Clever Sys Inc., Reston, VA, USA).

The tail suspension test revealed the animal’s depression-like behavior. The mouse was suspended by their tails with adhesive tape in a position about 1 cm away from the tail tips. The test was recorded for 6 min and the last 5 min were analyzed. The animal’s immobility was recorded when the mouse completely stopped any initiated movements.

### 2.11. Cognition Functional Tests

The Y-maze test was employed to reveal spatial working memory. The testing mouse was placed in a Y-shaped maze with three arms and given 10 min of free exploration. The arm entries and the sequence of entries by the mouse were recorded and counted. The ratio of spontaneous alternations (entering a different arm in each of three consecutive arm entries) was calculated to inspect spatial memory. Results are summarized as % Alternation = (Number of Alternations/[Total number of arm entries − 2]) × 100.

The Morris water maze test is regarded as a gold standard for the rodent’s spatial learning and working memory. Because of the stressful condition of forced swimming, this test was conducted as the final functional test in this investigation. The test was performed in a round pool filled with water; a hidden platform was placed 1 cm below the water surface. Mice received 5 consecutive training days to locate the platform, and the time spent was measured. Memory retention was inspected on the probe trial day when the platform was removed, and the animal was given 60 s to swim in the pool. The time and distance traveled by the testing mouse in the quadrant where the platform existed were recorded and measured using the TopScan Realtime Option Version 3.0 (Clever Sys Inc.).

### 2.12. Statistical Analysis

We performed statistical analysis using the data processing Prism 7 software (GraphPad Inc., Boston, MA, USA). According to the Power analysis using this software, based on variables in infarct formation, mortality, gene expression, and responses to interventions, experiments typically included 9–12 animals per group to yield a Power of ~90%. Animals were randomly assigned to experimental groups. The researcher who did the drug treatment and who analyzed data did not share the animal group information. For comparison between two groups, we used the Student two-tailed *t*-test. Multiple comparisons were performed using one-way or two-way ANOVA followed by Tukey’s correction. In graphical figures, data are presented as the mean ± SEM. Significant difference is defined when the *p*-value is less than 0.05. We present the actual *p* values in figures or figure legends for detailed evaluation.

## 3. Results

### 3.1. Preconditioning Effects of Early MEM Treatment Against Ischemic Stroke in Different AD Mouse Models

**Figure 1:**
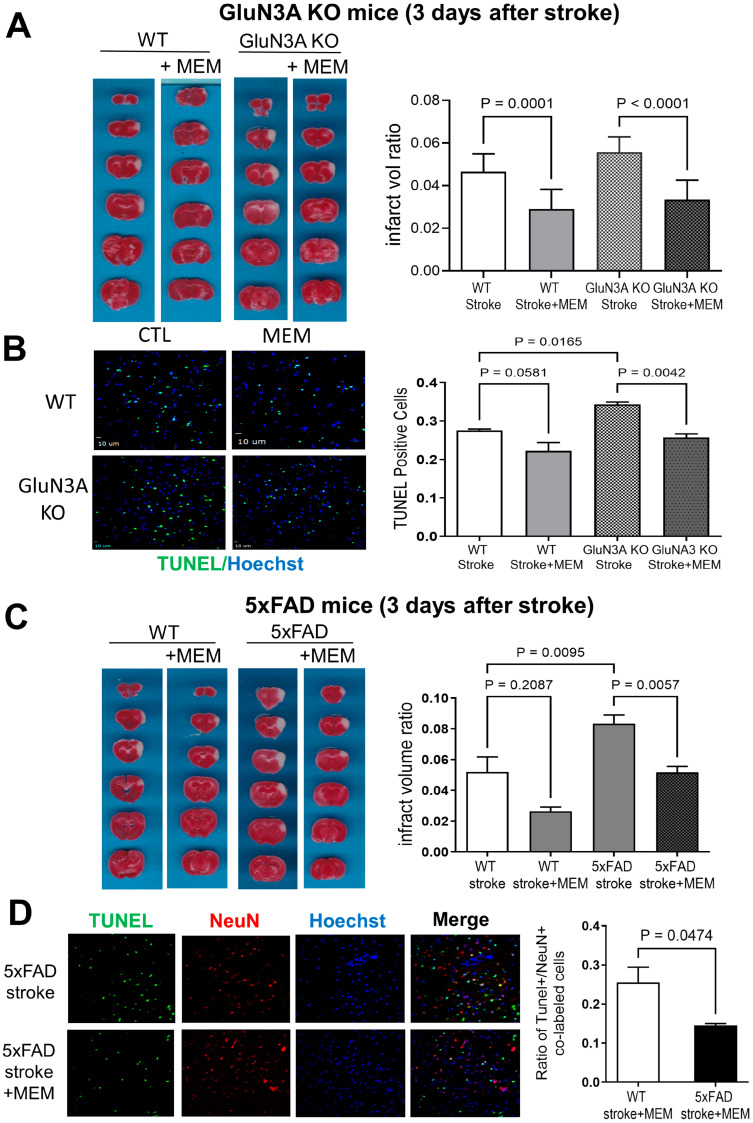
Preconditioning effects of early and chronic MEM treatment against ischemic stroke-induced brain injury in GluN3A KO mice and 5xFAD mice. Focal ischemia-induced infarct formation and neuronal cell death were assessed using the TTC staining and TUNEL staining, respectively. (**A**) TTC results of cortical infarction in brain sections of WT and GluN3A KO mice 3 days after focal ischemic stroke with and without MEM (10 mg/kg/day × 3 months). The quantified bar graph shows significant reductions in infarct volume induced by MEM in WT and GluN3A KO mice. N = 11 for WT stroke control and stroke plus MEM, respectively; n = 9 for GluN3A KO stroke and 8 for stroke plus MEM, respectively. Ordinary one-way ANOVA with multiple comparisons and Tukey post hoc corrections. Actual *p* values (*p* < 0.05) are shown in the figure (same for other figures). During the course of the investigation, there was 1 death in each of two WT stroke groups and 2 deaths in each of two AD stroke groups, indicating no clear link to MEM or surgery procedure. Dead animals were not included in the data analysis. (**B**) TUNEL immunoimaging assays of neuronal death in the peri-infarct region of the right sensorimotor cortex. The bar graph quantified the TUNEL-positive cells, showing significant decreases in these cells by MEM in WT and 5xFAD mice. N = 3 brains for each group. Ordinary one-way ANOVA with multiple comparisons and Tukey corrections. (**C**) Representative TTC images of cortical infarct areas in brain sections of a 5xFAD mouse and a WT control. The MEM treatment significantly reduced the infarct volume in these mice. (**D**) TUNEL staining of neuronal cell death in the peri-infarct region. Cell counting confirmed a significant neuroprotective effect, as evidenced by a decrease in TUNEL-positive cells in 5xFAD stroke mice treated with MEM compared to WT stroke mice. N = 3 brains in the WT and 5xFAD groups, respectively. Unpaired *t*-test; actual *p* values are shown in the bar graph.

The GluN3A KO mouse, 5xFAD mouse and their corresponding WT controls were treated with MEM (10 mg/kg/day in drinking water) or vehicle control for 3 months, starting at 3 months of age. After this, animals underwent surgery for focal cerebral ischemia. This surgery involved ligating the MCA’s distal branches supplying the right sensorimotor cortex [48]. The ischemic insult resulted in the formation of a local infarction, characterized by massive neuronal cell death in the core region 1–3 days later (Figure 1A,C). Three days after the stroke surgery, the infarct volume was measured using the TTC staining of brain sections, and cell death was inspected using a TUNEL staining kit. Compared to their WT controls, the infarct formation showed a trend of or a significant increase in the GluN3A KO brain and the 5xFAD brain, respectively (Figure 1A,C). The quantified analysis of TUNEL-positive cells revealed significantly increased cell death in both GluN3A KO mice and 5xFAD mice (Figure 1B,D). For AD mice which received MEM, ischemia-induced brain damage was significantly minimized, showing a marked anti-stroke preconditioning effect of the MEM pretreatment (Figure 1).

**Figure 2:**
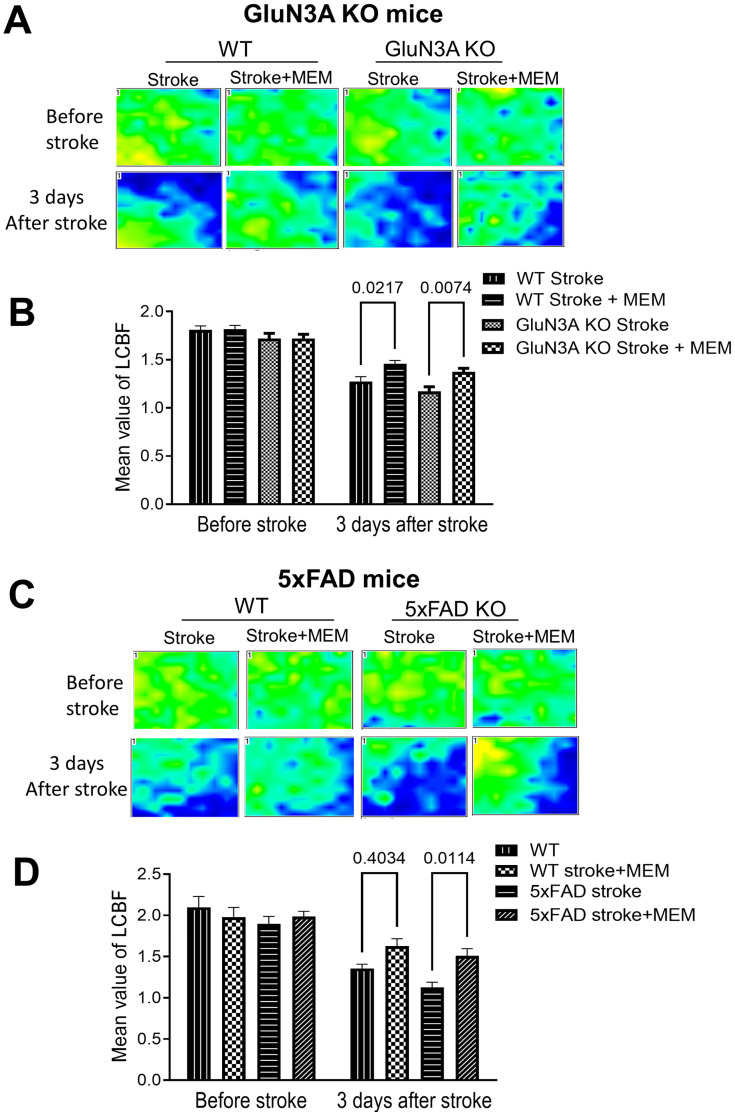
Improved local cerebral blood flow by MEM 3 days after ischemic stroke in GluN3A KO mice and 5xFAD mice. A laser Doppler scanner was used to measure LCBF in the peri-infarct region of the ischemic cortex 3 days after stroke. (**A**,**B**) In the GluN3A KO mouse brain, a significant decrease in LCBF was observed in the emerging area, indicated by the blue color. WT and GluN3A KO mice treated with MEM showed fewer blue areas, indicating vascular protection and enhanced flow recovery. The quantification analysis in B confirmed a moderate but significant increase in LCBF in the post-stroke AD mice. N = 12 per group. Two-way ANOVA with multiple comparisons and post hoc Tukey corrections. The actual significant *p* values are shown in the bar graph. (**C**,**D**) LCBF measured by the laser Doppler scanner in the peri-infarct region 3 days after stroke in the 5xFAD mouse. MEM similarly increased the local flow in the post-stroke 5xFAD brain. N = 12 per group. Two-way ANOVA with multiple comparisons and post hoc Tukey corrections. The actual significant *p* values are shown in the figure.

To further understand the preconditioning effect of MEM, LCBF was measured using a laser Doppler scanner after the ischemic insult. In the peri-infarct region of the stroke brain, measured 3 days after stroke, LCBF was drastically dropped compared to no-stroke animals (Figure 2A,C). The local flow was higher in the brains of WT and AD mice that received the MEM pretreatment (Figure 2B,D). This result implicated a protective effect of MEM on vascular integrity against ischemic injury [51].

**Figure 3:**
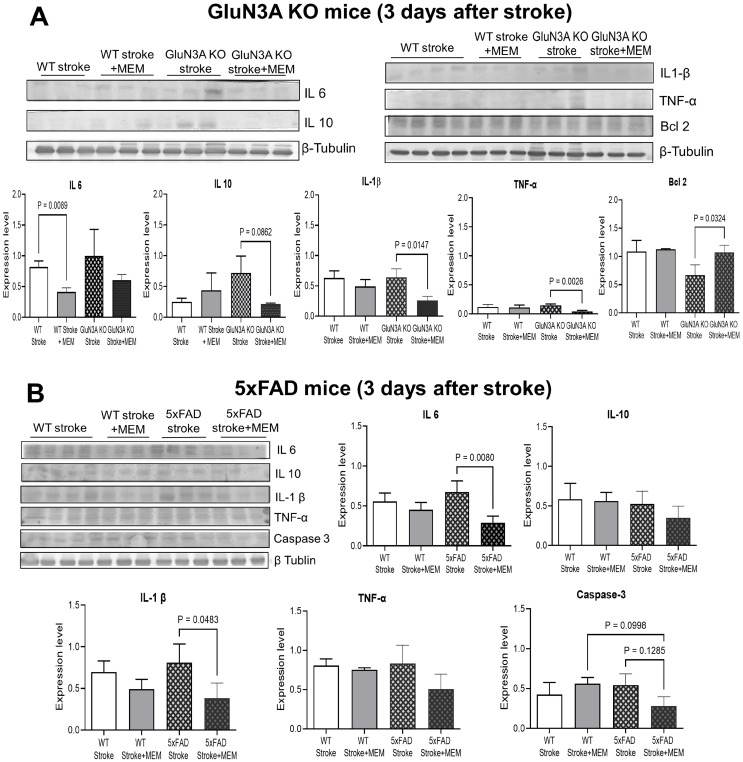
Regulation of inflammatory factors and apoptotic signals by MEM 3 days after ischemic stroke in AD brains. Western blot analysis of the protein levels of some inflammatory factors and apoptotic genes in the post-stroke AD brains. Baseline expression of these factors in no-stroke brains is presented in Supplemental Scheme S1. (**A**) In the cortical tissue of GluN3A KO mice, IL-1β, IL-6, IL-10 and TNFα were significantly attenuated in the MEM groups of post-stroke WT and/or GluN3A KO mice. The anti-apoptotic gene bcl-2 was significantly increased compared to no-MEM mice. N = 4, 3, 3 and 3 brains in WT, WT + MEM, GluN3A KO and GluN3A KO + MEM groups, respectively. Ordinary one-way ANOVA with multiple comparisons and post hoc Tukey corrections, significant *p* values are shown in the figure. (**B**) Western blotting analysis of inflammatory factors and the apoptotic gene caspase-3. Inflammatory factors IL-1β and IL-6 were significantly reduced in the MEM-treated brain. MEM showed a significant suppressing effect on the activated caspase-3, consistent with an anti-apoptotic effect. N = 4, 3, 3, 3 in WT, WT + MEM, 5xFAD and 5xFA + MEM groups, respectively. Ordinary one-way ANOVA with multiple comparisons and post hoc Tukey corrections, significant *p* values are shown in the figure.

Western blotting was used to inspect the expression of inflammatory and related factors in the peri-infarct region. Typically, the basal levels of these factors in non-stroke GluN3A KO adult brains did not change significantly (Supplemental Scheme S2). In the GluN3A KO brain three days after stroke, inflammatory factors IL-1β, IL-6, IL-10, and/or TNFα were significantly elevated, while MEM largely prevented these increases (Figure 3A,B). Meanwhile, MEM significantly enhanced the expression of the anti-apoptotic gene Bcl-2 (Figure 3A). In 5xFAD stroke mice, MEM showed similar effects or trends of decreasing these inflammatory factors, which should be neuroprotective [52]. In addition, MEM reduced the activation of apoptotic caspase-3 (Figure 3B), which was consistent with the decrease in TUNEL-positive cells and an anti-apoptotic effect.

**Figure 4:**
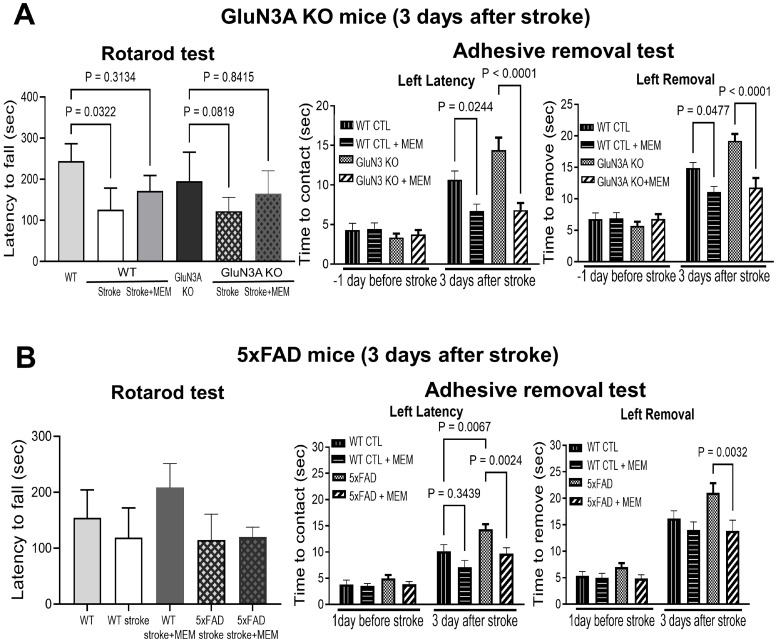
Effects of MEM on functional and psychological alterations 3 days after ischemic stroke in different AD mice. Locomotion and psychological deficits were inspected in sub-acute post-stroke AD mice. (**A**) In the rotarod test of locomotion activity and deficits developed in WT and GluN3A KO mice. The MEM treatment improved the time spent on the rotating cylinder, so the performance of stroke 5xFAD mice was comparable to that of their no-stroke controls. N = 4, 4, 5 for WT no-stroke control, WT stroke, and WT stroke + MEM, respectively; n = 8 for each 5xFAD group. Ordinary one-way ANOVA with multiple comparisons and post hoc Tukey corrections. Before the stroke, all animal groups performed similarly in the time to contact and time to remove the dot in the adhesive dot removal test. The ischemic insult to the sensorimotor cortex caused significant delays in these responses. Post-stroke GluN3A KO mice in the MEM group performed significantly faster than the no-MEM group. N = 4, 5, 5 for WT, WT stroke and WT stroke + MEM groups; n = 8 for each GluN3A KO group. (**B**) Locomotion and sensorimotor functions of 5xFAD mice 3 days after stroke. In the rotarod test, MEM did not improve locomotion in these animals. N = 5, 7, 3 in WT, WT stroke, and WT stroke + MEM groups, respectively. Two-way ANOVA with multiple comparisons and post hoc Tukey corrections. In the adhesive dot removal test, the sensorimotor function was largely protected by the MEM treatment, with significantly faster reaction and dot removal from the forepaws. N = 11 in each WT group; n = 12 in each 5xFAD group. Two-way ANOVA with multiple comparisons and post hoc Tukey corrections.

The ischemic stroke caused locomotion deficits. In the rotarod test 3 days after stroke, stroke mice fell off the rotating cylinder much sooner compared to WT controls (Figure 4A). In both WT and 5xFAD stroke groups, MEM treatment exhibited a trend of prolonging the time spent on the cylinder (Figure 4A,B). In the adhesive dot removal task one day before stroke, all animals quickly sensed and removed the stick dot from their front paw, because of the intact cortical-peripheral neuronal connections. Three days after stroke, the animals took longer to feel, contact, and remove the dot, a syndrome of deteriorated sensorimotor neuronal pathway (Figure 4A,B). GluN3A KO and 5xFAD mice showed even worse performance after stroke than WT stroke mice (Figure 4A,B). MEM treatment improved the perception and removal of the stick dot in these AD mice (Figure 4A,B).

### 3.2. Sustained Effects of MEM Chronic Treatment in Aging Post-Stroke AD Mice

**Figure 5:**
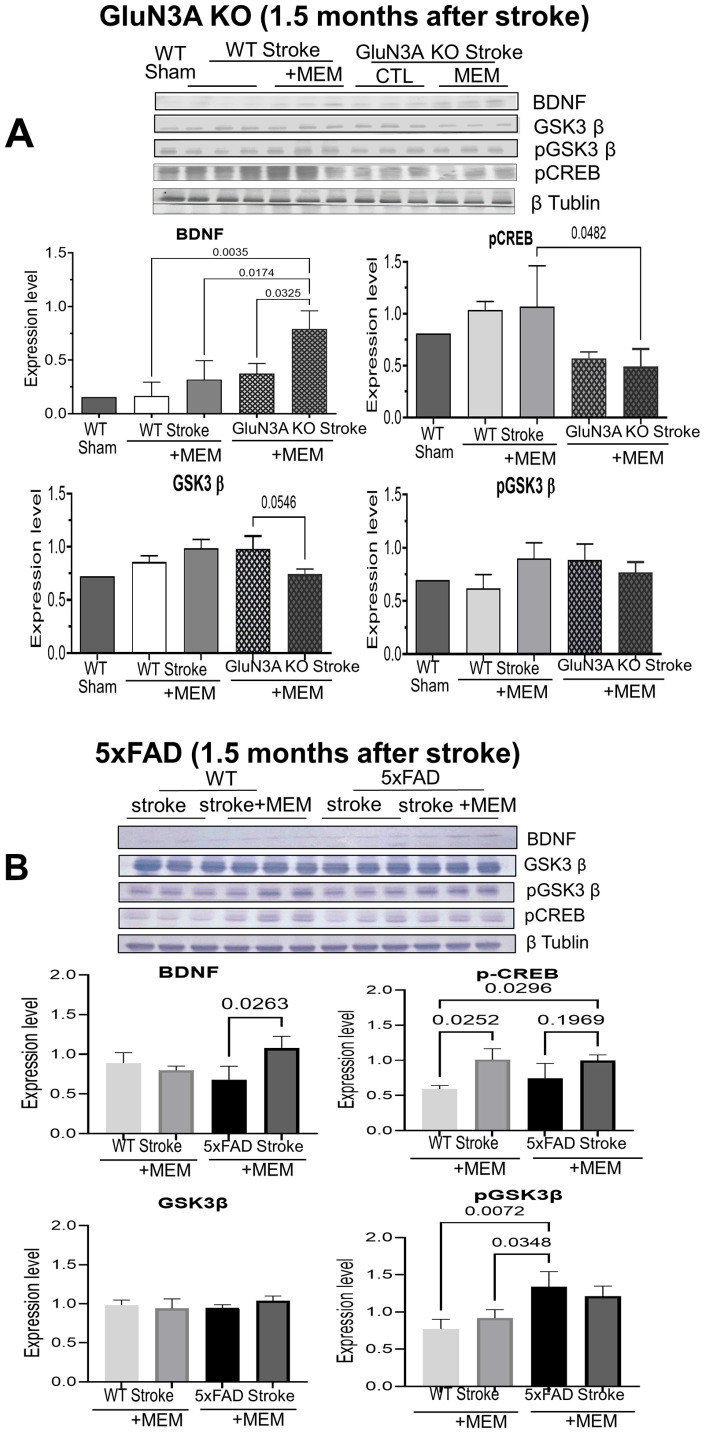
Long-term effects of MEM treatment on BDNF/GSK/CREB signaling in post-stroke AD brains. Western blot analysis examined key signaling proteins in the protective and regenerative BDNF/CREB ad GSK pathways 1.5 months after stroke in GluN3A KO and 5xFAD mice. In MEM groups, the drug was administered continuously before and after the stroke. (**A**) In the cortex of the post-stroke GluN3A KO mice that received MEM, the BDNF expression was significantly higher than in all other mouse groups. Somehow unexpected, the pCREB expression in the post-stroke GluN3A KO brain remained unchanged. MEM inhibited GSK3 expression in the GluN3A KO brain and showed a trend toward decreased pGSK3, suggesting anti-apoptotic and possibly other survival activities. N = 3 brains in each group, ordinary one-way ANOVA with multiple comparisons and post hoc Tukey corrections. (**B**) Effects of MEM in the post-stroke 5xFAD cortex. As in the GluN3A KO mouse, chronic MEM treatment increased BDNF expression and consistently elevated pCREB levels. MEM did not show any effect on GSK3β levels; meanwhile, pGSK3β increased in the 5xFAD cortex. The increased pGSK3β could play a pathological role in the 5xFAD brain. N = 3 in each group, ANOVA with multiple comparisons and post hoc Tukey corrections. In the long-term study, 2 WT stroke/aging mice in the non-MEM group died, and 3 died in the AD stroke/aging groups (2 and 1 without and with MEM, respectively); dead animals were not included in data analysis.

In separate animal groups, WT and AD mice continued to receive daily MEM treatment after stroke. Western blotting of the GluN3A KO brain in the MEM group showed a significant increase in BDNF expression, after 1.5 months of continued post-stroke MEM treatment (Figure 5A). BDNF release/activation initiates CREB phosphorylation and activation. CREB is a transcriptional factor involved in neuronal survival, synaptic plasticity, and memory function [53]. The activated form of phosphorylated CREB (pCREB) was ~50% lower in the GluN3A KO brain compared to WT mice (Figure 5A), which could contribute to neurodegeneration and cognition deficits in this AD model. MEM had no significant effect on the low pCREB expression (Figure 5A). In the 5xFAD mouse brain, the BDNF expression in MEM-treated brains significantly increased after stroke (Figure 5B). MEM enhanced the expression of pCREB in the post-stroke brains of either the WT or the 5xFAD mouse (Figure 5B). There was no difference in the total GSK3β level. Meanwhile, pGSK3β was higher in the 5xFAD brain than in WT brains (Figure 5B), suggesting a pathological role (see Supplemental Scheme S3 for additional verification data).

**Figure 6:**
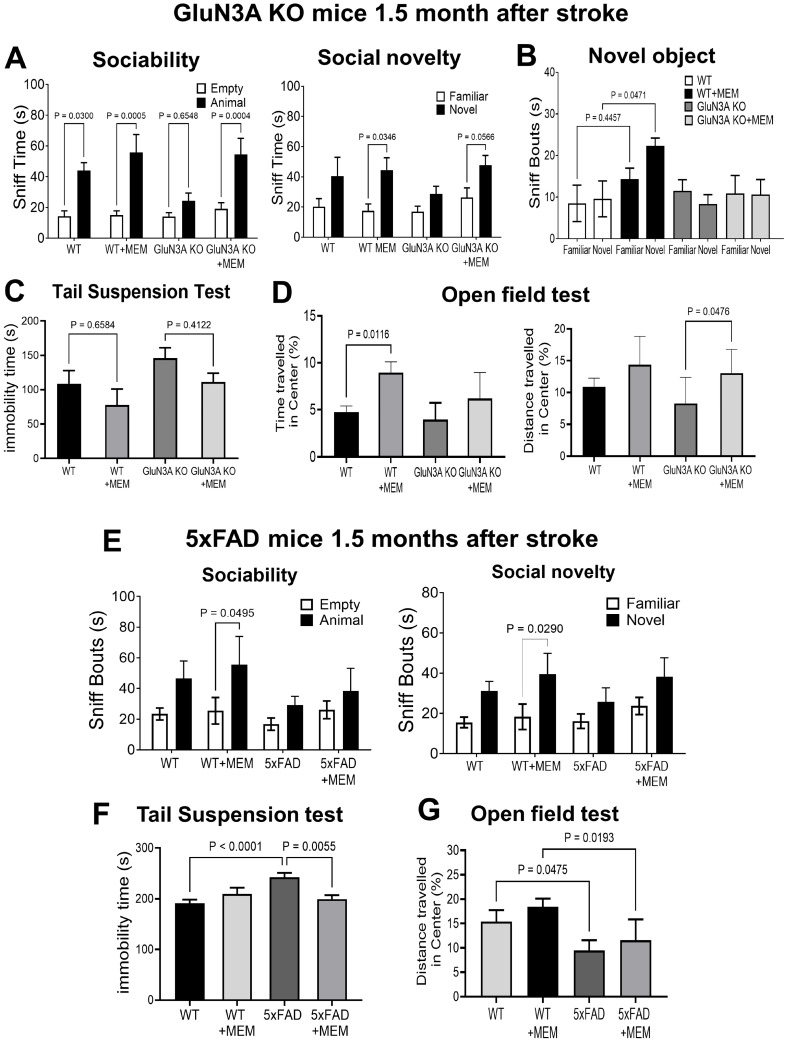
Long-term effects of MEM on social and psychological behaviors of post-stroke AD mice. Functional and behavioral benefits were assessed 1.5–2.0 months after stroke in animals that received continuous MEM or vehicle control. (**A**) In the three-chamber sociability test, WT mice before stroke displayed a preference for sniffing a partner. This preference disappeared in post-stroke GluN3A KO mice, whereas MEM maintained sociability at the level of normal controls. In the social novelty behavior, the MEM treatment effectively maintained the normal behavioral response of increased sniffing on a novel partner compared to a familiar one. N = 7 and 8 for WT and WT + MEM groups, n = 10 and 11 for GluN3A KO and GluN3A KO + MEM groups, respectively. Ordinary one-way ANOVA with multiple comparisons and post hoc Tukey corrections. (**B**) The novel object test. MEM notably increased the sniffing behavior in post-stroke WT mice. Other groups did not show significant alterations. Same animal numbers in each group as in (**A**). (**C**) The tail suspension test demonstrated that MEM significantly decreased the immobility time of WT and GluN3A KO mice, showing reduced depression in these chronically post-stroke animals. The same animal numbers as in (**A**). (**D**) In the open field test of anxiety behaviors, MEM promoted WT and GluN3A KO mice in terms of time and distance traveled in the center area, indicating attenuated anxiety. The same number of animals as in (**A**). (**E**) Social behavior three-chamber test in post-stroke 5xFAD mice. In the sociability and social novelty examinations, MEM helped maintain WT mice’s normal preference for a partner or a novel partner; however, this preference was not significant in 5xFAD mice. N = 5 for WT and 5xFAD group, respectively; ordinary one-way ANOVA with multiple comparisons and post hoc Tukey corrections. (**F**) The tail suspension test revealed that 5xFAD stroke mice suffered more depression-like behavior compared to WT stroke mice, and MEM significantly decreased this psychological behavior. The same animal number and statistical method were used as in (**E**). (**G**) Consistent with the psychological change, the open field test revealed that post-stroke 5xFAD mice exhibited a greater decrease in traveling in the central area. In the 5xFAD group that received MEM, the travel distance showed no significant difference in comparison to no-MEM 5xFAD mice (two-way ANOVA).

In functional assays, continuous pre- and post-stroke MEM treatment significantly improved social activity in post-stroke mice. In the three-chamber test, GluN3A KO mice lost the increased sniff activity of social behavior, while MEM kept the social preference toward the partner (Figure 6A). Similar effects were seen in the social novelty test. MEM restored the increased interest in a novel partner (Figure 6A). In the novel object test, post-stroke WT mice failed to differentiate between familiar and novel objects. The MEM benefit was not seen in 5xFAD mice (Figure 6B).

In the tail suspension test, WT and GluN3A KO post-stroke mice treated with MEM showed trends of reducing immobility (Figure 6C). In the open field test of WT post-stroke mice, MEM significantly improved the travel time in the central zone. In GluN3A KO post-stroke mice, MEM improved travel distance in the central area (Figure 6D), indicating a reduction in anxiety behavior. MEM corrected social deficits of post-stroke WT mice but not 5xFAD mice (Figure 6E). The tail suspension and open field tests suggested that MEM improved psychological behaviors in post-stroke 5xFAD mice and their WT controls. (Figure 6F,G).

**Figure 7:**
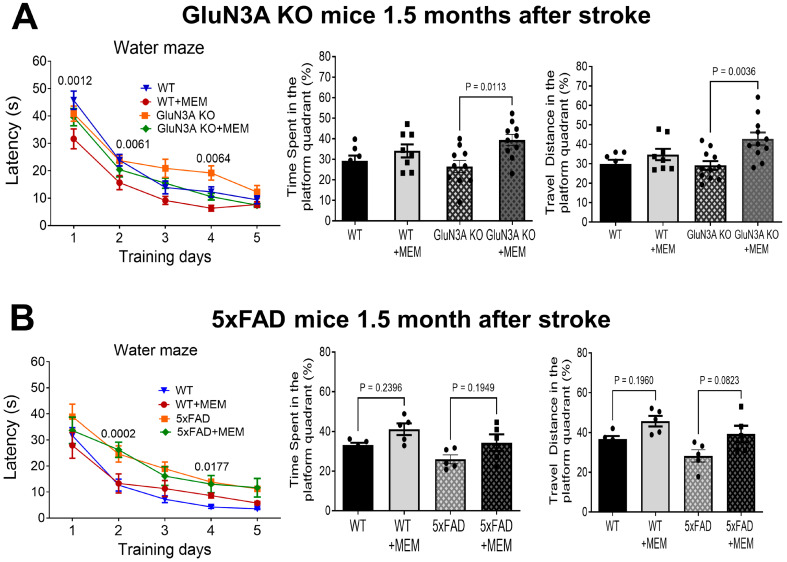
Long-term benefits of MEM on the cognitive function of post-stroke AD mice. The Water maze test was conducted to inspect learning and memory function 1.5 months after stroke (7.5 months old) of GluN3A KO mice and 5xFAD mice. (**A**) Animals were subjected to 5 training days to identify the location of a platform where they could stay away from swimming in the water. On day one, the post-stroke WT mice in the MEM group performed the best, with the shortest time to locate the platform, significantly faster than stroke WT mice without MEM. Similar deference was observed on day two: WT mice receiving MEM spent significantly less time than WT mice without MEM. On day 4, GluN3A KO mice in the MEM group were substantially faster to locate the platform than GluN3A KO mice without MEM. In probe trials conducted 1 day after training, GluN3A KO mice in the MEM group showed better memory for the platform location, spending significantly longer time swimming in the platform quadrant. In WT mice, MEM appeared to improve memory, but this trend was not statistically significant. In the swimming distance assessment in the platform quadrant, GluN3A KO mice receiving MEM showed the best spatial memory; however, the effect of MEM in WT mice was not statistically significant. N = 7 and 8 mice for WT and WT + MEM group, respectively; n = 11 and 11 for GluN3A KO and GluN3A KO + MEM group, respectively. Two-way ANOVA with multiple comparisons and post hoc Tukey corrections. (**B**) During the five training days, post-stroke WT mice with or without MEM generally learned faster than post-stroke 5xFAD mice, resulting in distinctive learning curves, particularly during the first three days. In the probe task for spatial memory in WT and 5xFAD mice, MEM showed a trend toward increased time spent in the platform area, although this difference was not statistically significant. In the swimming distance analysis, MEM-treated post-stroke WT and 5xFAD mice traveled significantly farther in the platform area, indicating enhanced spatial memory. N = 7 and 8 mice for WT and WT + MEM group, respectively; n = 11 and 11 for 5xFAD and 5xFAD + MEM groups, respectively. Ordinary one-way ANOVA with multiple comparisons and post hoc Tukey corrections.

During the 5-day training of the Morris water maze test, MEM-treated WT and GluN3A KO mice displayed enhanced learning capability. Escape latency was shorter for these mice, particularly on days 1 and 2 for WT mice, and day 4 for GluN3A KO mice (Figure 7A). After training, probe trials of memory retention revealed that MEM-treated GluN3A KO mice spent significantly longer time and traveled more in the hidden platform quadrant (Figure 7A). 5xFAD mice with stroke and their WT controls had distinct learning curves during training. MEM did not affect the learning curve of post-stroke 5xFAD mice and WT controls. Probe trials showed that MEM-treated WT and 5xFAD mice spent more time in the targeted platform quadrant, with increased travel distance (Figure 7B). These results suggest that chronic treatment with MEM improved post-stroke psychological behaviors and learning/memory functions. The MEM effects, however, appeared to be variable and multifaceted, presumably due to various cellular and molecular mechanisms in different AD models.

**Figure 8:**
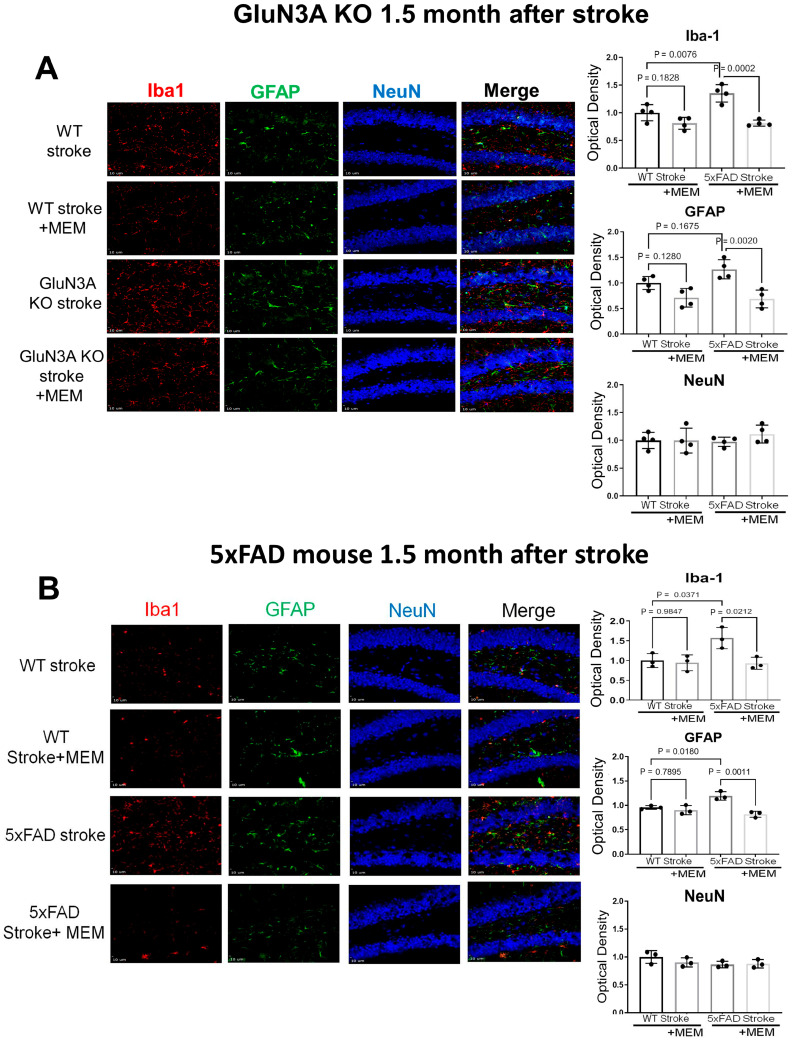
Suppression of MEM on inflammatory glial cells in post-stroke AD brains. Immunohistochemical staining was conducted on brain sections to inspect microglia cells, astrocytes, and neuronal cells in GluN3A KO mice and 5xFAD mice 1.5 months after stroke. (**A**) Representative immunofluorescent images of iba-1, GFAP- and NeuN-positive cells in the hippocampus of chronic post-stroke GluN3A KO mice. As expected, in the neurodegenerative brain, there were noticeable Iba-1-positive activated microglia and GFAP-positive reactive astrocytes; MEM significantly suppressed the number of these cells in both the WT and GluN3A KO brains. NeuN-positive neuronal cells were unaltered, which is consistent with the ischemic insult that selectively targeted the sensorimotor cortex with no direct impact on the hippocampus. N = 3 brains per group, one-way ANOVA with multiple comparisons and post hoc Tukey corrections. (**B**) Representative fluorescent images of iba-1, GFAP, and NeuN staining in the brain sections of 5xFAD mice and their WT controls. Activated microglia and astrocytes were significantly suppressed by the MEM treatment in the chronically post-stroke AD brains. There was no significant change in the number of NeuN-positive cells. N = 3 brains per group, one-way ANOVA with multiple comparisons and post hoc Tukey corrections.

Immunohistochemical examinations of the post-stroke hippocampal brain sections revealed increased Iba-1-positive microglia cells and GFAP-positive astrocytes (Figure 8). This cellular response was mostly suppressed in MEM-treated mice (Figure 8). The NeuN staining did not detect significant alterations of neuronal cells in the hippocampus, which was expected in this stroke model, where the ischemic insult does not target the hippocampus.

**Figure 9:**
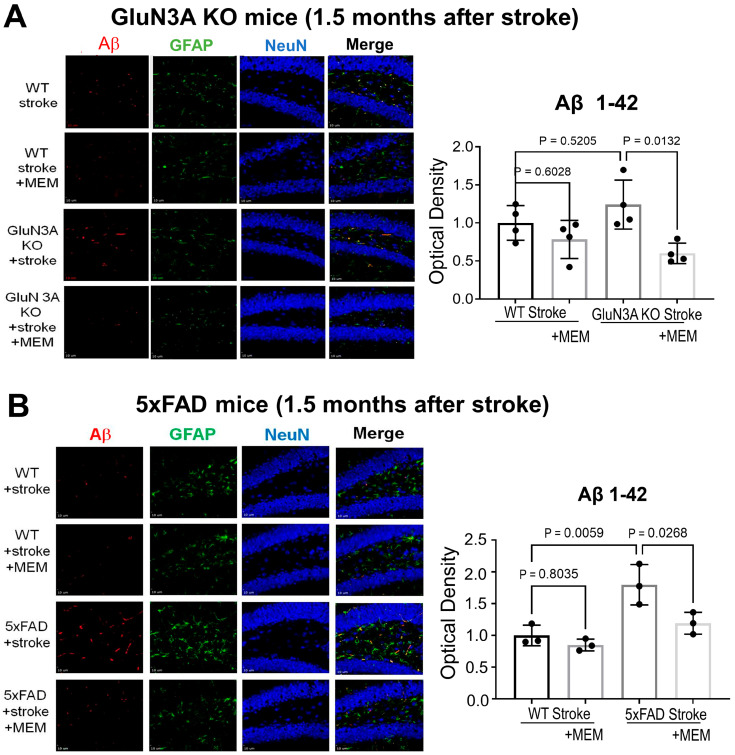
Suppression of MEM on amyloid deposition in post-stroke AD brains. The amyloid peptide Aβ 1–42 (Aβ42) deposition in the hippocampus of GluN3A KO mice and 5xFAD mice was examined using immunohistochemical staining 1.5 months after stroke with and without continual MEM treatments. (**A**) Representative immunofluorescent images of the Aβ42 level in the hippocampus of chronic post-stroke GluN3A KO mice. The Aβ42 staining mostly overlapped with GFAP-positive astrocytes (orange color in the merged image) and some NeuN-positive neurons (purple color in the CA1/2 region). The bar graph on the right quantified the optical density of all Aβ42 expression, showing a significant reduction in this amyloid peptide in the post-stroke hippocampus of the WT + MEM group and the GluN3A KO + MEM group. N = 3 brains per group. Ordinary one-way ANOVA with multiple comparisons and Tukey corrections. (**B**) Similar Aβ42 deposition and MEM effects were observed in the post-stroke 5xFAD brain. The amyloid accumulation, however, in the 5xFAD brain appeared to be twice that in the WT brain. The MEM treatment could significantly attenuate the Aβ42 deposition.

We previously showed that MEM had a noticeable effect of reducing Aβ accumulation in the no-stroke AD brain [17]. In the current study, Aβ42 deposition in the hippocampus of 5xFAD stroke mice was doubled compared to post-stroke WT controls (Figure 9A). MEM treatment in 5xFAD mice significantly decreased Aβ42 deposition in the hippocampus (Figure 9B).

## 4. Discussion

This investigation tested the concurrent preventive and preconditioning effects against the comorbidity of AD and stroke in two AD mouse models. The GluN3A KO mouse represents a distinct pathogenesis of the NMDAR subunit GluN3A deficiency as a sporadic AD model based on the Ca^2+^ hypothesis; the 5XFAD mouse is regarded as a typical transgenic human amyloid pathology as an FAD model based on the amyloid hypothesis. The experimental design of testing different AD models was to validate and improve the clinical significance of the proposed MEM preventive therapy. In the absence of exogenous FAD gene expression, GluN3A deficiency spontaneously induces age-dependent early and late syndromes similar to those observed in human patients, including olfactory deficits, psychological disorders, sensorimotor impairments in young and adult individuals, and cognitive decline in middle-aged adults. Notably, significant Aβ42 and tau pathology emerge in the cortex and hippocampus after, but not before, dementia in GluN3A KO mice, implicating an amyloid-independent AD pathogenesis [17]. On the other hand, the 5xFAD mouse is a widely tested and accepted FAD/AD model. The AD phenotype of the 5xFAD mouse and other similar models primarily relies on an artificial expression or manipulation of FAD-associated genes [54,55]. They are suitable and effective for proving the toxic effects of amyloid, tau, and other AD-related genes. We noticed that many of these models only partly mimic clinical symptoms of AD patients [54,55] while the GluN3A KO mouse develops most late-onset AD syndromes [17].

The chronic MEM treatment began at 3 months of age, which is the preclinical stage in these AD models. To imitate strokes occurring to over 50% of AD patients, a focal ischemic stroke was induced in AD mice at 6 months old. Compared with vehicle groups, the MEM treatment exhibited significant protective and preconditioning efficacy against AD progression and acute ischemic brain injury. Inspections of mice receiving MEM identified increased levels of trophic factors, decreased neuroinflammation/apoptosis, and significantly improved psychological and cognitive functions. According to this study, early intervention targeting NMDAR hyperactivity could protect aging individuals with AD and stroke risk factors with multiple benefits. Further research is needed to understand how MEM works in a disease-specific manner to achieve dual benefits. This includes exploring how MEM and similar drugs affects both acute and chronic excitotoxicity at molecular, cellular and neural network levels, as well as their impacts on structural and functional improvements.

In clinical treatments and trials, almost all so-called “early” and “preventive” therapies are conducted at the prodromal stage of MCI or the dementia stage [36,56]. MEM is currently only used as a symptomatic treatment for advanced AD patients. The decision was based on the belief that NMDAR abnormalities are merely a consequence of Aβ/tau pathology [56,57]. However, this judgment contradicts growing evidence, including results from this investigation. NMDAR and neuronal hyperactivity are common in the early stages of AD development and persist throughout disease progression [15,16,17,24]. Based on the modified Ca^2+^ hypothesis of AD proposed from our investigation, NMDAR overactivation and chronic Ca^2+^ dysregulation often occur as an early primary event in an Aβ-independent manner [4,17,36]. It can be assumed that the marginal and sometimes inconsistent efficacy of current MEM treatment is primarily due to improperly delayed treatment. MEM administered to the late-stage AD patients misses the pathogenic phase of neuronal hyperactivity that occurs and persists for years during the preclinical stage. As shown in the present investigation, we believe a genuine game-changing preventive therapy should be applied as early as possible, before noticeable amyloid/tau pathology and cognitive decline [4,36]. The identification of risk factors and mutant genes through human genome investigations, the development of early biomarkers, and the advancement of imaging technologies have enabled a preemptive approach for susceptible individuals [58,59,60].

BDNF induction is suppressed by eNMDAR activation [61]. BDNF attenuates cognitive deficits in AD mice [62]. The MEM-induced BDNF expression in the AD brain is an important effect and can act as an essential mediating mechanism for the neuroprotective and preconditioning benefits. BDNF release from astrocytes regulates activation of neuronal populations via the TrkB receptor [63]. This functional benefit of BDNF is independent of improvements in either Aβ or tau pathology [62], suggesting a BDNF-triggered, AD/tau-independent mechanism. The BDNF-CREB signaling pathway plays a crucial role in neuroplasticity, neuroprotection, and cognitive function. Activated CREB (pCREB) serves as a downstream regulator of gene expression involved in neuronal survival, synaptic plasticity, and various cognitive processes, including learning and memory [53]. The increasing effect of MEM on the pCREB levels is the first evidence in post-stroke AD mice. This highlights a potential treatment for AD and ischemic stroke. Endorsing these possibilities, viral delivery of CREB-binding protein (CBP) increases BDNF expression and improves cognitive function in an AD model without affecting Aβ or tau pathology [64].

Previous and recent research has referred to NMDAR-mediated excitotoxicity as the primary cell death mechanism in ischemic stroke and other brain injuries [10]. Generating novel and safe NMDAR antagonists and regulatory reagents targeting the glutamate system remains a high priority in neuroscience research and drug development [10,65]. Selective eNMDAR antagonists such as MEM are preferred choices to minimize side effects while enhancing the neuroprotective effects [4,66]. MEM is a low-affinity, uncompetitive, and use-dependent NMDAR antagonist with unique voltage- and Mg^2+^-dependent properties, acting at moderate membrane depolarizations [67]. Unlike typical NMDAR antagonists, MEM targets excitotoxicity-associated extrasynaptic GluN2B- and GluN2C/2D-containing NMDARs. This is especially the case when used at therapeutic doses (1–10 µM in vitro and 1–30 mg/kg in vivo). Clinical trials have consistently demonstrated the safety of MEM (1–30 mg/kg/day) for short- and long-term uses, with an adverse event profile “similar to that of placebo” [68,69]. High doses of MEM (e.g., ≥30 mg/kg) should be avoided due to the potential for sNMDAR block, which can lead to neuronal loss and impairments. This dose-dependent side-effect is not a surprise considering most other drugs.

A variety of factors and shortcomings in preclinical and clinical research can contribute to failures of NMDAR antagonists in clinical stroke trials. One critical dilemma is the narrow therapeutic window. For an NMDA receptor antagonist to be protective, it must be given soon after the ischemic attack. This is often difficult to do in practice [70]. Unlike acute neuroprotective treatments, preconditioning with sublethal ischemia, hypoxia, or various chemicals/drugs can enhance the tolerance of animal and human brains, including neuronal and non-neuronal cells, to impending severe ischemic or hypoxic insults [71,72]. The preconditioning benefits of MEM, as demonstrated in this investigation in AD mice, provide a unique and valuable opportunity for a clinically viable application of preconditioning therapy.

It is important to note that, among stroke survivors, physical impairments improve after stroke, but cognitive impairments always progressively worsen [73]. Clinically significant, even a minor stroke can have a profound impact on daily functioning, including the ability to make executive decisions and maintain cognitive capabilities [74]. PSD-like disorders have also been detected in mouse ischemic stroke models, including the focal cortical stroke model in our investigations [48,75,76]. Our recent investigation revealed that mice developed psychological/psychiatric disorders 4 to 8 weeks after stroke. In the chronic phase after stroke, the non-ischemic prefrontal cortex (PFC) showed impaired neuronal plasticity. This included suppressed LTP, reduced BDNF and oxytocin signaling, and disturbed dopamine synthesis/uptake [48]. Although the cortical ischemic insult does not directly impact the hippocampus [77], these animals develop impaired hippocampal synaptic plasticity and hippocampus-dependent spatial memory loss 3–6 weeks after the stroke. These observations support the use of MEM treatment to protect synaptic and cognitive integrity in AD mice and patients. More research is needed to understand how focal ischemia in the cortex causes cognitive impairments and interacts with AD progression. This could lead to a deeper understanding of AD mechanisms and the development of new therapies.

## 5. Conclusions and Future Direction

Acute and chronic excitotoxicity is seen in ischemic stroke and neurodegenerative diseases [78,79]. The decisive role of NMDARs in the pathological event positions these receptors as major molecular, cellular, and network players in brain functions and pertinent therapeutic targets [80]. Future research of retrograde assessments on the stroke prevalence and outcomes in MCI/AD/ADRD patients with or without chronic MEM treatments may provide clinical evidence for the dual efficacy of MEM. Preclinical trials of the MEM preventive therapy are needed. Meanwhile, strategies that help to maintain Ca^2+^ homeostasis are worth testing in the preclinical stage. The interaction between NMDARs and TRPMs as a downstream signaling pathway to excitotoxicity provides another potential target for therapies against the comorbidity of stroke and AD/ADRD [4].

A limitation of this study is the test of only male mice, while females have a significantly higher incidence of AD than males. Moreover, there are evident sex-dependent changes in the glutamate system, especially in NMDARs, both in physiological [81] and pathophysiological conditions [82]. A further investigation should verify the reported observation and explore possible gender differences, including the underlying mechanisms, to improve the accuracy and efficacy of clinical applications.

A growing consensus suggests that Aβ pathology may not be the primary pathogenic mechanism in sporadic AD [26,27,28,29]. Understanding the causal roles of amyloid peptides, glutamatergic hyperactivity, and downstream cascades is crucial. This knowledge, in both Aβ-dependent and -independent ways, will shed light on the disease development over the years/decades preceding MCI/AD diagnosis. It will encourage novel ideas and promote the development of effective therapies for the comorbidity of AD/ADRD, ischemic stroke, and PSD, which are increasing clinical cases affecting people with defective genes and/or neurodegenerative risk factors.

## Data Availability

The original contributions presented in this study are included in the article/Supplementary Material. Further inquiries can be directed to the corresponding author.

## Supplementary Materials

The following supporting information can be downloaded at: https://www.mdpi.com/article/10.3390/cells14231871/s1, Scheme S1: A diagraph sketch showing the timeline of the experimental design. GluN3A KO and 5xFAD mice were tested in this investigation, starting around the young adult age of 3-months old. Each AD model was compared to their corresponding WT/non-stroke controls. MEM (10 mg/kg/day in drinking water) was administered for 3 months. Behavioral/functional assessments were performed before and/or after a focal cortical ischemic stroke. Brain injury and LCBF were inspected 3 days after stroke; more animals were continuously tested for another 1 to 1.5 months before further functional and pathological examinations; Scheme S2: Baseline levels of some key factors in non-stroke GluN3A KO and 5xFAD mice and their controls. In the cortical tissue of non-stroke mice of 6-month old, inflammatory and other factors were assessed using Western Blotting. In Glu3A KO mice, all factors except p-CREB were not significantly different from age-matched WT controls. 5xFAD mice, otherwise, displayed significantly increased GSK3b signaling and the APOE level. Their p-CREB and BDNF levels were noticeably low compared to WT controls. The protein levels of IL-6, IL-10 and TNFa were virtually undetectable, no bar graph was generated. N = 3 independent brain samples/factor; * *p* < 0.05, ** *p* < 0.01 vs. corresponding controls. One-way ANOVA with post-hoc test; Scheme S3: Additional evidence for alterations of signaling pathways in GluN3A KO and 5xFAD mice Additional Western blotting analysis of a few key signal proteins in WT and AD/stroke mice with and without MEM treatments. The trends of the MEM effect were consistent with the examination shown in Figure 5.
