# Supplementary material for: Combined Preventive and Preconditioning Treatments for the Comorbidity of Alzheimer’s Disease and Ischemic Stroke in a GluN3A Knockout Mouse and a 5xFAD Mouse
Source: Cells. 2025 Nov 26;14(23):1871. doi: 10.3390/cells14231871 (PMC12691033; doi:10.3390/cells14231871)
Supplement: Supplementary file 1 [file cells-14-01871-s001.zip › cells-3929298-supplementary.pdf]

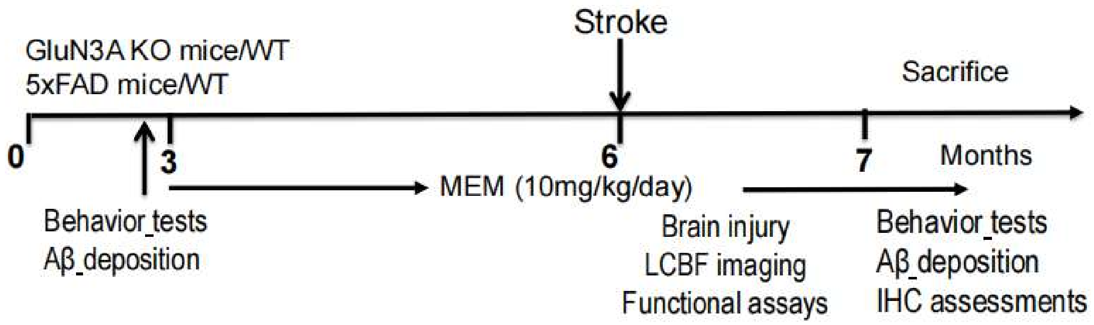

Scheme 1. A diagraph sketch showing the timeline of the experimental design. GluN3A KO and 5xFAD mice were tested in this investigation, starting around the young adult age of 3-months old. Each AD model was compared to their corresponding WT/non-stroke controls. MEM (10 mg/kg/day in drinking water) was administered for 3 months. Behavioral/functional assessments were performed before and/or after a focal cortical ischemic stroke. Brain injury and LCBF were inspected 3 days after stroke; more animals were continuously tested for another 1 to 1.5 months before further functional and pathological examinations. .

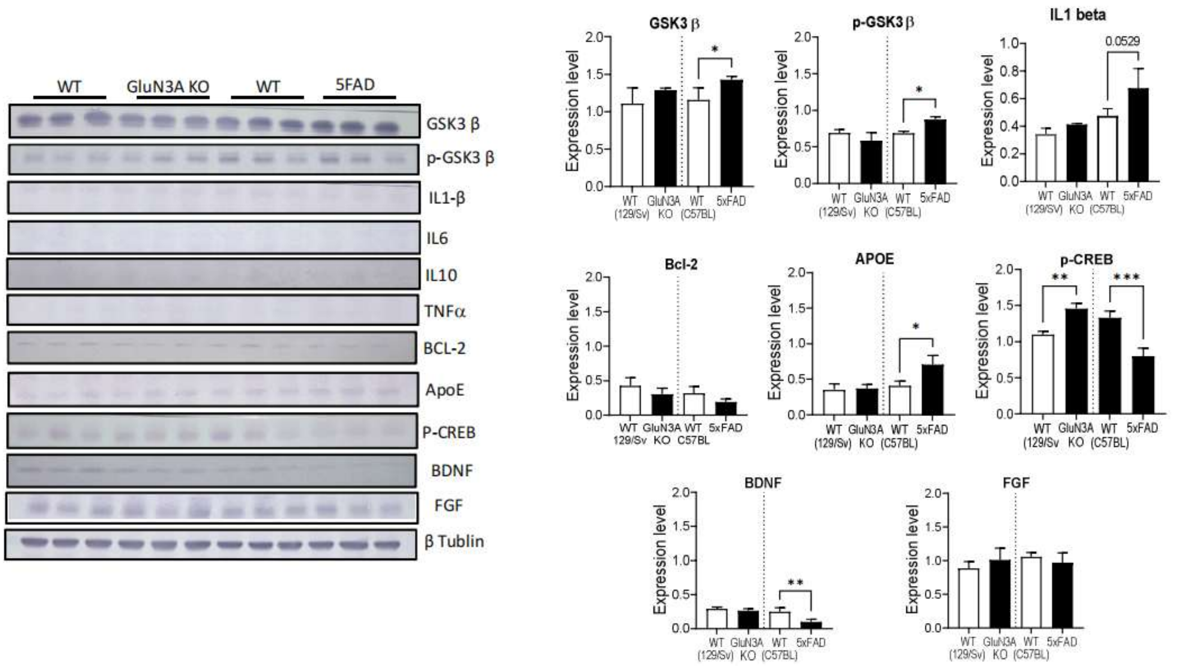

Scheme 2. Baseline levels of some key factors in non-stroke GluN3A KO and 5xFAD mice and their controls. In the cortical tissue of non-stroke mice of 6-month old, inflammatory and other factors were assessed using Western Blotting. In Glu3A KO mice, all factors except p-CREB were not significantly different from age-matched WT controls. 5xFAD mice, otherwise, displayed significantly increased GSK3b signaling and the APOE level. Their p-CREB and BDNF levels were noticeably low compared to WT controls. The protein levels of IL-6, IL-10 and TNFa were virtually undetectable, no bar graph was generated. N=3 independent brain samples/factor; *$p<0.05$, **$p<0.01$ vs. corresponding controls. One-way ANOVA with post-hoc test.

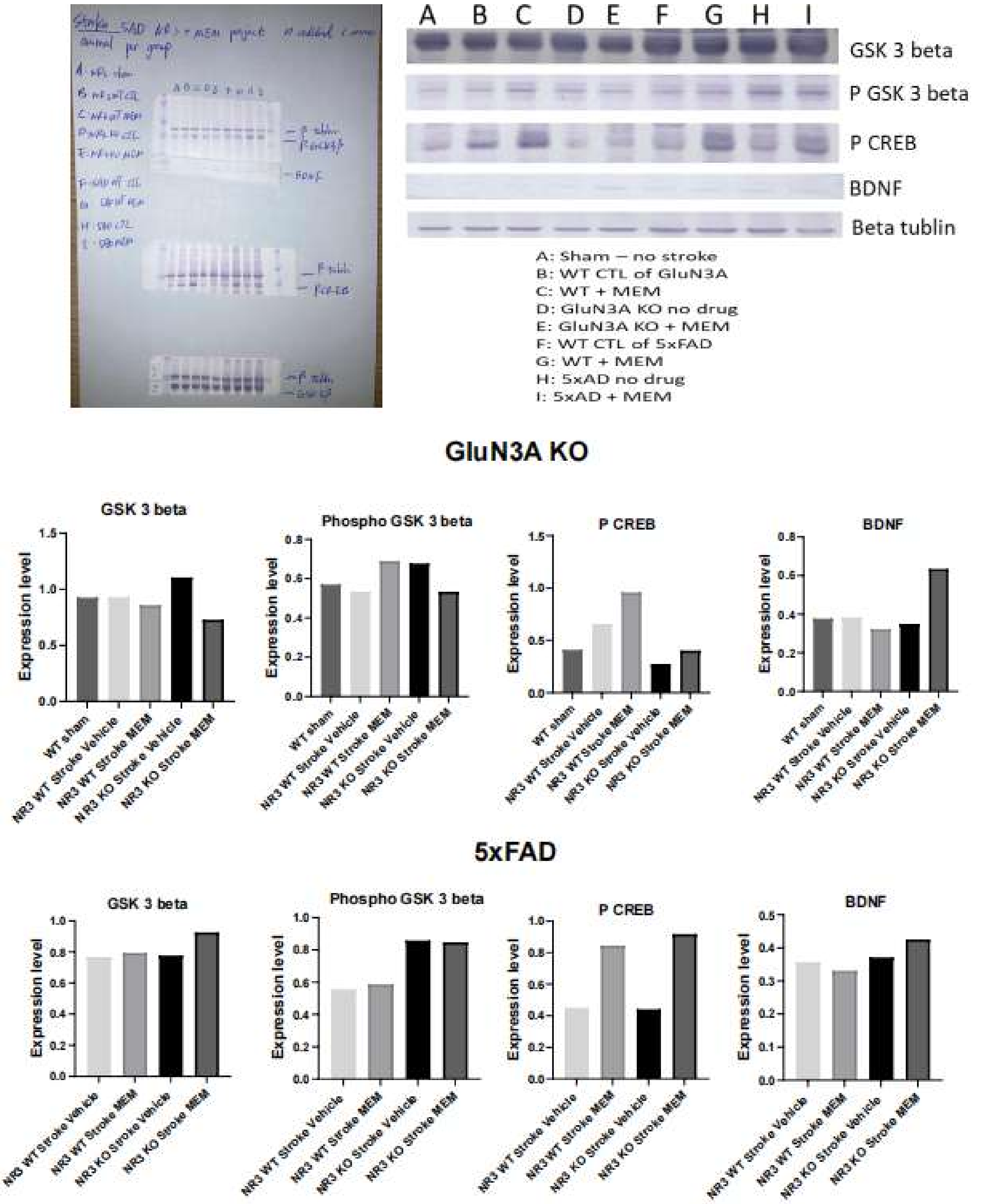

Scheme 3. Additional evidence for alterations of signaling pathways in GluN3A KO and 5xFAD mice Additional Western blotting analysis of a few key signal proteins in WT and AD/stroke mice with and without MEM treatments. The trends of the MEM effect were consistent with the examination shown in Figure 5.
